# Protein–Phenolic Interactions as a Factor Affecting the Physicochemical Properties of White Bean Proteins

**DOI:** 10.3390/molecules24030408

**Published:** 2019-01-23

**Authors:** Łukasz Sęczyk, Michał Świeca, Ireneusz Kapusta, Urszula Gawlik-Dziki

**Affiliations:** 1 Department of Industrial and Medicinal Plants, University of Life Sciences in Lublin, 15 Akademicka Street, 20-950 Lublin, Poland; 2 Department of Biochemistry and Food Chemistry, University of Life Sciences in Lublin, 8 Skromna Street, 20-704 Lublin, Poland; michal.swieca@up.lublin.pl (M.Ś.); urszula.gawlik@up.lublin.pl (U.G.-D.); 3 Department of Food Technology and Human Nutrition, Rzeszów University, 4 Zelwerowicza Street, 35-601 Rzeszów, Poland; ikapusta@univ.rzeszow.pl

**Keywords:** phenolic compounds, food proteins, interactions, protein–phenolic complexes, physicochemical properties, white bean, albumins, globulins

## Abstract

This study was conducted with an aim to determine the interactions of pure phenolic compounds (gallic acid, ferulic acid, chlorogenic acid, quercetin, apigenin, and catechin) and phenolics from plant extracts (green tea and green coffee) with protein fractions of white bean (albumins and globulins). The physicochemical properties of complexes were established through an analysis of the UV-Vis spectrum; relative content of free amino groups, thiol groups, and tryptophan residues; chromatographic (SE-HPLC) and electrophoretic (SD-PAGE, Native-PAGE) properties; and conformational changes reflected by Fourier transform infrared spectra. Further, the effect of pH and ionic strength on the solubility and stability of complexes as well as the binding capacity of phenolics to proteins were determined. Results show that, in most cases, phenolics significantly affected the measured parameters; however, the effects were strongly differentiated by the type of phenolic compounds and protein fraction that were applied. Moreover, it may be that changes in the properties of complexes are reflected in the biological nature of proteins and phenolic compounds such as their bioavailability and physiological activity. However, due to the structural complexity of proteins, and the multitudinous factors that affect their interactions, such studies are a great and long-term challenge for the domain of food science.

## 1. Introduction

Phenolic compounds are a widely distributed group of secondary plant metabolites [1,2,3]. They are synthesized by plants to protect themselves from environmental stress factors, such as ultraviolet radiation or aggression by pathogens [2,4]. This group of phytochemicals is characterized by phenolic structural features—they contain several hydroxyl groups on one or more six-carbon aromatic rings [5,6]. According to the number of aromatic rings and other structural elements (e.g., hydroxyl and carboxyl groups) that bind these rings to one another, these phytochemicals may be divided into five main classes: flavonoids, phenolic acids, phenolic alcohols, stilbenes, and lignans [1,6,7].

Phenolic compounds are widespread components of plant-derived foods, including fruits, vegetables, cereals, olive, legumes, chocolate, tea, coffee, and wine [1,2,5]. As food components, these compounds affect the bitterness, astringency, color, flavor, odor, and oxidative stability of food products [8]. Additionally, they exhibit many favorable biological activities, of which antioxidant properties are those that are most thoroughly studied [1,2,7].

Phenolics are considered to be one of the most effective antioxidants in the human diet due to their multifactorial mechanism of action. These substances are able to inhibit the formation of lipid radicals, disrupt the propagation of chain auto-oxidation reactions, suppress singlet oxygen, reduce hydrogen peroxides to stable compounds, chelate transition metal ions, inhibit endogenous prooxidative enzymes, and activate endogenous antioxidant enzymes [9]. Therefore, they are potential agents for the prevention of various degenerative diseases associated with oxidative stress, especially cancers, cardiovascular diseases, diabetes mellitus, osteoporosis, and neurodegenerative diseases [2,3,7].

Given their health-promoting properties, phenolics are commonly used as natural additives for improving the pro-health status of many basic food products such as bread and pasta [10,11]. Nevertheless, recent studies have shown that the incorporation of phenolic compounds to food products modify their functional, nutritional, and nutraceutical properties. Some reports show that this effect may be the consequence of the interactions of phenolics with food matrix components such as proteins, carbohydrates, and lipids [12,13,14,15].

Proteins are essential nutrients for the human body, and their role in the human diet has been well established for a long time [16]. Due to the importance of phenolic compounds and proteins in nutrition and health, there is a growing interest in protein–phenolic interactions [15,16,17].

As previously described, the formation of protein–phenolic complexes may have a significant influence on protein structure, solubility, hydrophobicity, thermal stability, and the isoelectric point [15,17]. Furthermore, the binding of phenolic compounds to proteins results in the blocking of some amino acid residues [15,17,18,19]. Therefore, physicochemical changes caused by phenolic compounds may determine important biological properties, including the digestibility and utilization of food proteins as well as the activity of digestive enzymes. Conversely, phenolic complexation with proteins may also influence the bioaccessibility and activity of phenolics [15,17,18,19].

Taking into account the significant role of proteins and phenolics in health, nutrition, and quality of food products as well as the widespread interest in the interaction of food components, we attempted to determine the effect of selected phenolic compounds on the physicochemical properties of proteins from the white bean as representative food proteins.

The purpose of this study was to show the interactions of pure phenolics (gallic acid [GA], ferulic acid [FA], chlorogenic acid [CGA], quercetin [Q], apigenin [A], and catechin [CAT]) and phenolics from plant extracts (from green tea [GT] and green coffee [GC]) with white bean protein fractions (albumins and globulins) to study the physicochemical properties of complexes, such as the protein UV-Vis spectrum; relative content of free amino groups, thiol groups, and tryptophan residues; chromatographic (SE-HPLC), electrophoretic (SD-PAGE, Native-PAGE); and spectral (Fourier transform infrared [FT-IR]) properties. Furthermore, the effect of pH and ionic strength on the solubility and stability of complexes as well as the binding capacity of proteins for phenolics were determined.

## 2. Results

### 2.1. UV-Vis Spectra of Protein–Phenolic Complexes

**Figure 1:**
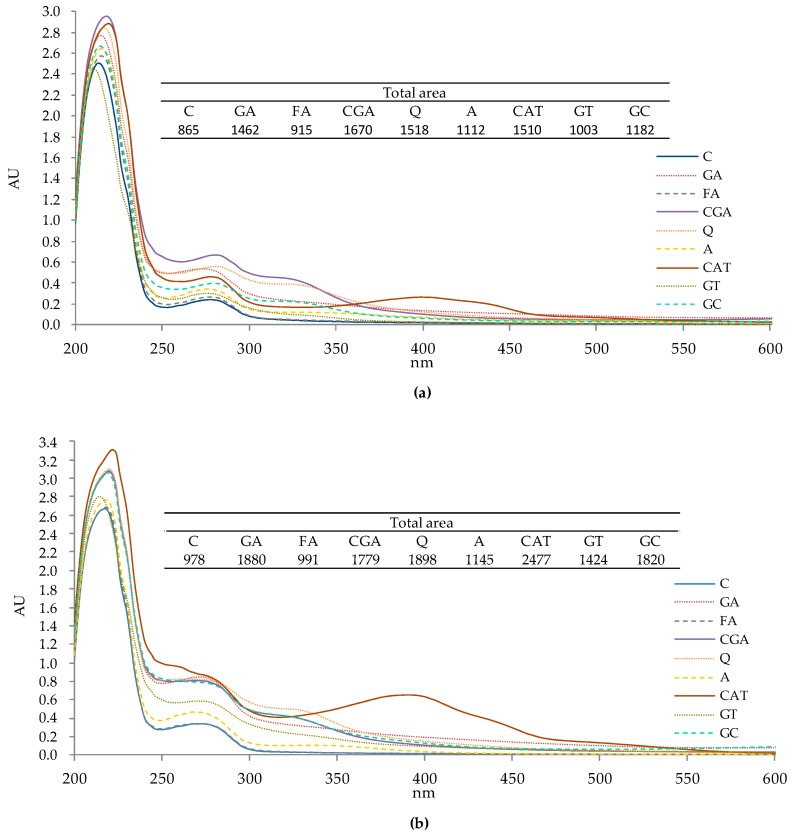
UV-Vis spectra of protein–phenolic complexes. (**a**) albumins; (**b**) globulins. C–control sample, GA, FA, CGA, Q, A, CAT, GT, GC–protein samples after incubation with gallic acid, ferulic acid, chlorogenic acid, quercetin, apigenin, catechin, green tea, and green coffee extracts, respectively.

The UV-Vis spectra of protein–phenolic complexes are presented in Figure 1. The results show that “pure” phenolic compounds, as well as phenolics from plant extracts, affected the UV-Vis spectra of white bean protein fractions. In case of both albumin (Figure 1a) and globulin (Figure 1b) fractions, total peak areas of protein samples treated by phenolics were significantly higher than those of the control, except for the sample treated by FA where the least pronounced effect was noted. Additional peaks in the wavelength range 300–350 nm were observed especially for CGA, Q, and green coffee extract (GC) complexes with maximum absorbance at approximately 325 nm. The incubation of protein fractions with CAT resulted in a significant increase of absorbance at 350–450 nm, with maximum absorbance at approximately 400 nm. Additionally, we noticed differences in absorption properties (shapes and areas) between albumin and globulin fractions, both for control and tested samples. Furthermore, it should be mentioned that the observed color of the protein samples changed after incubation with phenolic compounds; for example, the addition of CGA and green tea resulted in an olive green solution of protein, quercetin, and catechin resulted in a dark orange and light brown coloring of protein samples, respectively (results not presented).

### 2.2. Changes in Content of Free Amino Groups, Thiol Groups, and Tryptophan Residues of Proteins Affected by Phenolic Compounds

**Figure 2:**
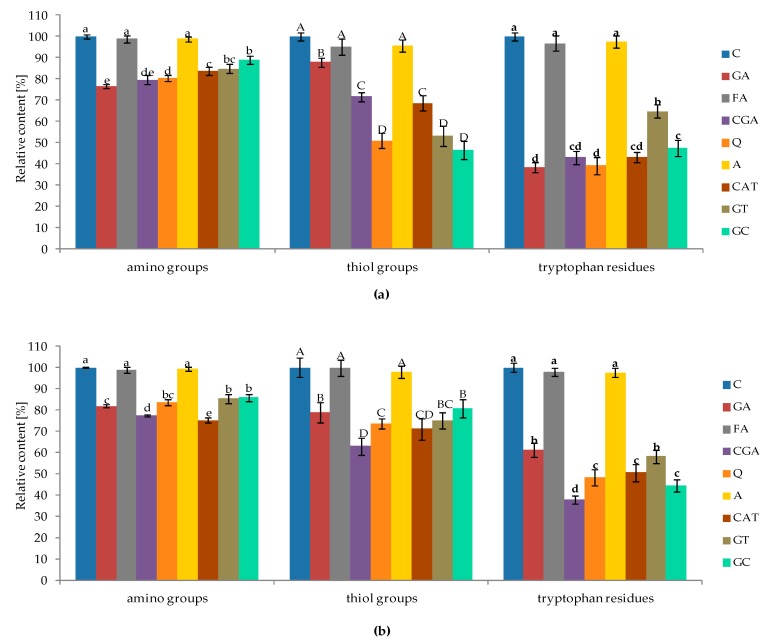
The effect of phenolic compounds on the relative abundance of free amino groups, thiol groups, and tryptophan residues of proteins: (**a**) albumins; and (**b**) globulins. Bars represent means ± SD. Means (*n* = 3), followed by different normal lowercase letters (a, b, c…) for amino groups, uppercase letters (A, B, C…) for thiol groups, and bold lowercase letters (a, b, c…) for tryptophan residues, in bars are significantly different at α = 0.05. C–control sample, GA, FA, CGA, Q, A, CAT, GT, GC–protein samples after incubation with gallic acid, ferulic acid, chlorogenic acid, quercetin, apigenin, catechin, green tea, and green coffee extracts, respectively.

The effect of phenolic compounds on the abundance of free amino groups, thiol groups, and tryptophan residues of protein fractions is illustrated in Figure 2 (a, albumins; b, globulins). In most cases, the addition of phenolic compounds to albumins and globulins resulted in a significant decrease in the amounts of their free reactive groups. However, a slight (insignificant) effect was observed only for protein fractions incubated with FA and A.

In case of albumins (Figure 2a), the most significant decrease in the amount of free amino groups was determined for GA (decrease by 23% in comparison to control). The highest negative impact on the abundance of free thiol groups was noticed for GC (decrease by 53%). However, there were no significant differences among GT, Q, and GC. The relative content of free tryptophan residues decreased significantly up to 62% after incubation with GA, and a similar impact was determined for Q, CGA, and CAT with which it decreased by 61%, 57%, and 57%, respectively.

The effect of phenolic compounds on the decrease in the content of reactive groups of albumins was observed to have the following order GA > CGA > Q > CAT > GT > GC > A > FA (free amino groups); GC > Q > GT > CAT > CGA > GA > FA > A (free thiol groups); and GA > Q > CAT > CGA > GC > GT > FA > A (tryptophan residues).

For globulins (Figure 2b), the maximal decrease of free amino groups was determined for CAT (decrease by 25% compared to control). The abundance of free thiol groups was reduced by up to 37% after CGA treatment. Additionally, CGA had the highest negative influence on the amount of free tryptophan residues—which decreased by 62%. In the case of globulins, the following order in the affinity of phenolic compounds to reactive sites of proteins were noticed: CAT > CGA > GA > Q > GT > GC > FA > A (free amino groups); CGA > CAT > Q > GT > GA > GC > A > FA (free thiol groups); and CGA > GC > Q > CAT > GT > GA > A > FA (tryptophan residues). Furthermore, both in the case of albumins and globulins, phenolic compounds had a more negative impact on the amount of free tryptophan residues, compared with the free amino and thiol groups.

### 2.3. Size-Exclusion High-Performance Liquid Chromatography (SE-HPLC)

**Figure 3:**
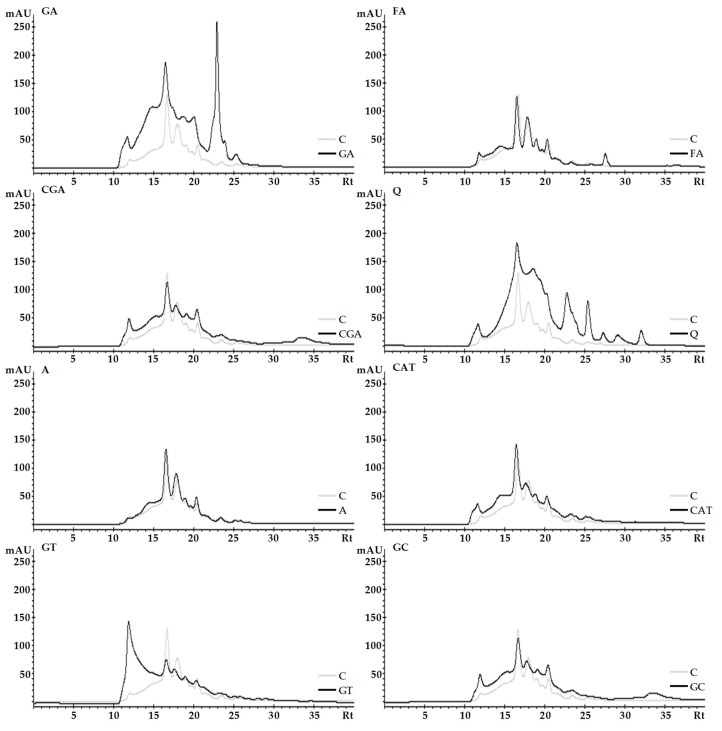
The effect of phenolic compounds on the SE-HPLC elution profiles of albumins. C–control sample, GA, FA, CGA, Q, A, CAT, GT, GC–protein samples after incubation with gallic acid, ferulic acid, chlorogenic acid, quercetin, apigenin, catechin, green tea, and green coffee extracts, respectively.

**Figure 4:**
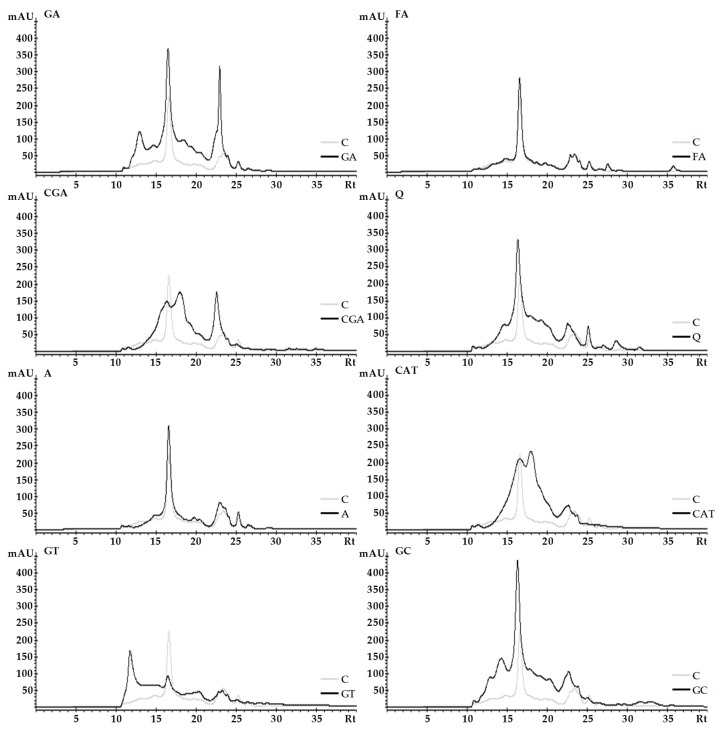
The effect of phenolic compounds on the SE-HPLC elution profiles of globulins. C–control sample, GA, FA, CGA, Q, A, CAT, GT, GC–protein samples after incubation with gallic acid, ferulic acid, chlorogenic acid, quercetin, apigenin, catechin, green tea, and green coffee extracts, respectively.

The effect of phenolic compounds on the SE-HPLC elution profiles of albumins and globulins is shown in Figure 3 and Figure 4, respectively. The results for albumins show that the chromatogram areas of proteins treated with phenolics were larger than those in the control sample (Figure 3). This effect was particularly pronounced for GA and Q complexes. Furthermore, changes in the size, shape, and retention time (Rt) of some individual peaks as well as the appearance of new peaks as a consequence of the addition of phenolics were found. An increase of individual peak size and a decrease of Rt compared to the control was determined for GA (e.g., peaks with Rt 16.5 and 23 min) among others. In comparison to the control, the most relevant changes in the shape of the chromatogram were detected for the GT sample—there was a significant increase of absorbance areas toward the lower Rt (10–16 min), which corresponded to an increase in the ratio of fractions with a higher molecular weight. For the globulin fraction (Figure 4), we found similar observations, such as a significant increase of chromatogram areas (especially for GA, Q, CAT, and GC), only a slight effect of FA and A on the SE-HPLC profile, as well as changes in the size, shapes, and Rt of some individual peaks. Moreover, in the case of the GT sample, an increase of the absorbance areas toward the lower Rt was observed (Figure 4). Furthermore, in the GT sample, some new peaks were noticed (e.g., for CGA and CAT samples, peaks with Rt 18 min).

### 2.4. Polyacrylamide Gel Electrophoresis

**Figure 5:**
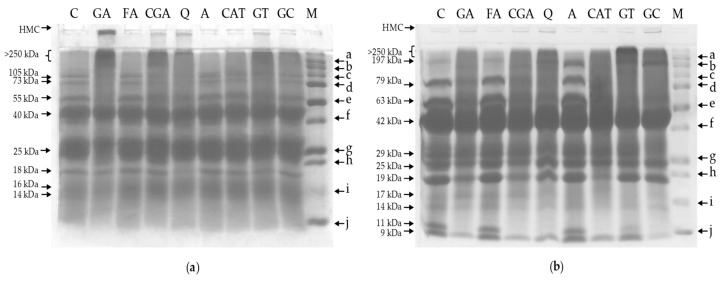
The effect of phenolic compounds on the SDS-PAGE profile of: (**a**) albumins; (**b**) globulins. C–control sample, GA, FA, CGA, Q, A, CAT, GT, GC–protein samples after incubation with gallic acid, ferulic acid, chlorogenic acid, quercetin, apigenin, catechin, green tea, and green coffee extracts, respectively; M–molecular weight markers: a (250 kDa), b (150 kDa), c (100 kDa), d (75 kDa), e (50 kDa), f (37 kDa), g (25 kDa), h (20 kDa), i (15 kDa), j (10 kDa); HMC (high molecular complex).

**Figure 6:**
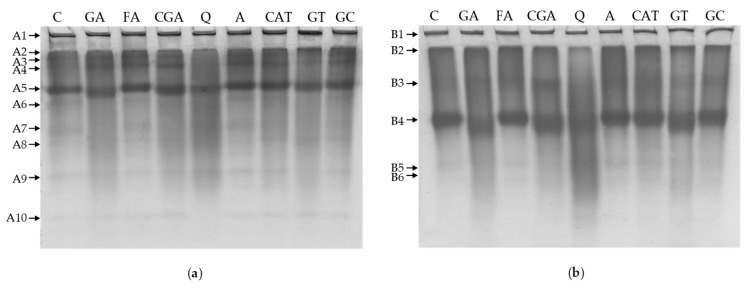
The effect of phenolic compounds on the Native-PAGE profile of: (**a**) albumins; (**b**) globulins. C–control sample, GA, FA, CGA, Q, A, CAT, GT, GC–protein samples after incubation with gallic acid, ferulic acid, chlorogenic acid, quercetin, apigenin, catechin, green tea, and green coffee extracts, respectively; A1–A10: bands detected for albumins, B1–B5: bands detected for globulins.

The effect of phenolic compounds on the electrophoretic patterns of white bean proteins is illustrated in Figure 5 and Figure 6. Protein fractions were analyzed both under reducing and denaturing conditions (SDS-PAGE; Figure 5) as well as without reducing and denaturing agents (Native-PAGE; Figure 6). Changes in the intensity of band staining (that reflect the relative abundance of a single protein fraction) and in band migration that were affected by most of the applied phenolics were observed for both albumin and globulin fractions.

In case of SDS-PAGE of albumins for the control sample, eight main bands that correlated with molecular masses of 105, 73, 55, 40, 25, 18, 16, and 14 kDa were detected (Figure 5a). The results show that the addition of phenolic compounds caused the formation of high molecular complexes (HMC; bands at the bottom of wells), which were most clearly visible for GA, CGA, Q, and GT samples. Additionally, we noticed the presence of complexes with molecular weight higher than 250 kDa (e.g., GA, CGA, GT). The abovementioned complexes (>250 kDa) were visualized in the form of smears below the interface between the stacking and resolving gels. Furthermore, compared to the control, reduction of the staining intensity of some bands was noticed. Blurry and fuzzy bands were detected; for example, for GA and Q samples, the bands correlated with molecular masses of 105, 73, and 55 kDa as well as 105, 73, and 18 kDa, respectively.

For the control sample of globulins, 11 main bands were identified (197, 79, 63, 42, 29, 25, 19, 17, 14, 11, and 9 kDa; Figure 5b). HMC occurred in the case of GA, CGA, Q, and GC samples. However, it was significantly less abundant than the albumins. Moreover, the addition of GT and GC extracts and Q to globulins resulted in the occurrence of bands >250 kDa. Compared to the control, a decrease in the staining intensity of some individual proteins such as bands that correlated with a molecular mass of 19 kDa for GA, CGA, and CAT samples as well as the absence of bands that correlated with a molecular mass of 11 kDa (control) for GA, CGA, Q, and CAT samples were also observed. Furthermore, in-lane smearing appeared for GA, CGA, Q, CAT, GT, and GC samples.

Results from Native-PAGE of albumins show that, for the control sample, ten main bands were detected (A1–A10; Figure 6a). In comparison to the control, the most relevant increase of the ratio of protein in band A1 was found for GT, which resulted in the highest staining intensity of the A1 band for this sample. On the other hand, a decrease in the staining intensity as well as blurring of some bands were observed. This effect was most prominent for Q (A2, A3, A5, and A9) and GT (A2 and A5). Moreover, phenolic compounds lead to slight changes in the migration of some bands. Particularly in the case of the A4 band in GA and CGA samples, we observed increased mobility.

In case of globulins, six main bands were detected for the control sample (B1–B6; Figure 6b). The incorporation of phenolic compounds into globulins generally had no prominent influence on the abundance of the B1 band. A slight effect was observed only for GT; however, it was not as significant as it was for albumins (GT; A1). In comparison with the control, we found a slight increase in the mobility of B4 bands for GA, CGA, Q, and GT samples and more intensive smearing in lanes for GA and Q. Among all of the phenolic compounds that were studied, the incubation of albumins and globulins with FA and A had the least effect on electrophoretic patterns (SDS-PAGE and Native-PAGE; Figure 5 and Figure 6).

### 2.5. Effect of pH and Ionic Strength on the Solubility of Protein–Phenolic Complexes

**Figure 7:**
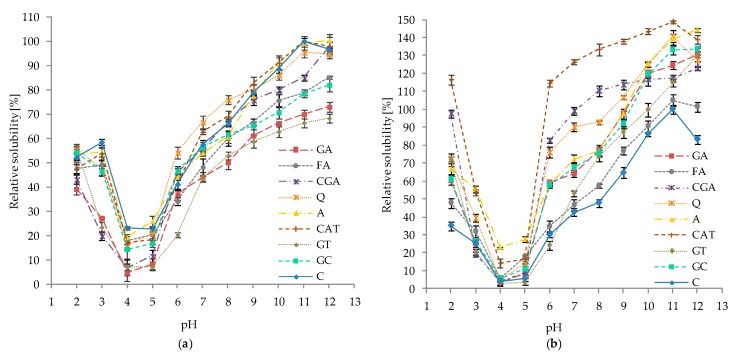
Relative solubility of white bean proteins affected by phenolic compounds at different pH values: (**a**) albumins; and (**b**) globulins. Points represent means (*n* = 3) ± SD. C–control sample, GA, FA, CGA, Q, A, CAT, GT, GC–protein samples after incubation with gallic acid, ferulic acid, chlorogenic acid, quercetin, apigenin, catechin, green tea, and green coffee extracts, respectively.

The solubility of protein fractions that were influenced by interactions with phenolic compounds at different pH (2–10) is shown in Figure 7 (a, albumins and, b, globulins).

As illustrated, the protein solubility depends on the pH and, in most cases, this property significantly changed among the phenolic compounds as compared to the control. In general, the solubility of albumins incubated with phenolics was lower than those for the control, which was most noticeable for GT and GA samples. A slight, but statistically significant, increase in the solubility of albumins affected by phenolics was observed for the Q sample in the pH range 6–8 (an increase of 9–10%) and the CAT sample in the pH range 7–9 (an increase of 4–6%). The highest negative impacts of phenolic compounds on solubility was observed: at pH 3—for GT (decrease by 35%), CGA (decrease by 32%), and GA (decrease by 31%); and at pH 11—for GT (decrease by 33%) and GA (decrease by 30%).

In contrast to the results obtained for albumins, phenolic compounds in general positively affected the solubility of globulins (Figure 7b). With regard to the control, a slight decrease of protein solubility was determined only for GT in the pH range 3–6 (decrease of up to 6% for pH 6). Among all of the studied samples, the most significant increase was noticed for the globulin fraction treated with CAT. Furthermore, in this case, solubility was higher in the entire measured pH range (2–12); however, the most prominent effect was noticed at pH 6, 7, and 8—an increase of 85%, 84%, and 86%, respectively.

**Figure 8:**
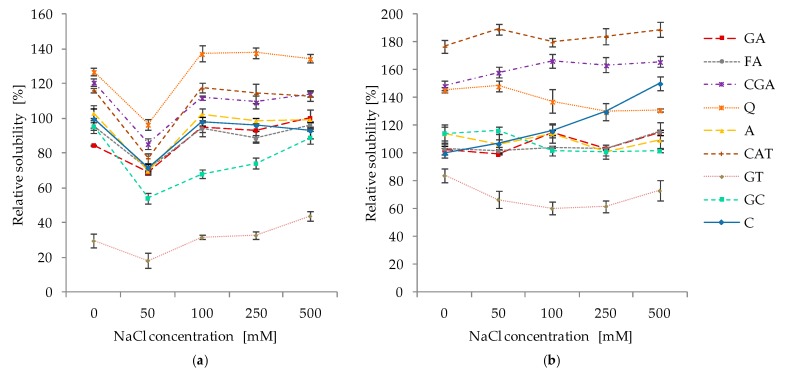
Relative solubility of white bean proteins affected by phenolic compounds at different ionic strengths: (**a**) albumins; and (**b**) globulins. Points represent means (*n* = 3) ± SD. C–control sample, GA, FA, CGA, Q, A, CAT, GT, GC–protein samples after incubation with gallic acid, ferulic acid, chlorogenic acid, quercetin, apigenin, catechin, green tea, and green coffee extracts, respectively.

The effect of ionic strength on the solubility of protein–phenolic complexes is presented in Figure 8. As expected, the incorporation of phenolic compounds into proteins influenced solubility at different ionic strengths. In case of albumins (Figure 8a), for all samples, the lowest solubility was determined at a slight ionic strength (50 mM NaCl), which was significantly lower than that at other ionic strengths (0 mM NaCl, 100–500 mM NaCl). In the case of C, FA, A, and CAT samples, an increase of ionic strengths in the range of 100–500 mM NaCl had no significant effect on further protein solubility changes. However, in the abovementioned range for CGA and GC samples, a significant decrease in solubility was determined in comparison to native conditions (0 mM NaCl). In contrast, the solubility of GA, Q, and GT increased in the range of 100–500 mM NaCl. Among all of the samples, the lowest solubility was noticed for GT, especially for the sample treated with 50 mM NaCl, where it was only 18% of that recorded for the control sample under native conditions. Nevertheless, a further increase of ionic strength (100–500 mM NaCl) resulted in an increase in the GT solubility of up to 43% of that for the control sample. Among all of the samples, the highest solubility was determined for Q, where it was higher by up to 34% that was noticed in the presence of 250 mM NaCl.

For the control sample of globulins, the solubility increased along with ionic strength; however, a significant increase was observed only for higher concentrations of NaCl (250–500 mM; Figure 8b). A similar solubility in comparison with control samples at all tested ionic strengths was observed for FA and A. The incubation of proteins with CAT and CGA resulted in a significant increase of protein solubility as compared to the control at all studied ionic strengths. For example, the solubility of the CAT sample was higher than that for the control sample in native conditions (0 mM NaCl) and up to 80% in the presence of 500 mM NaCl. Similarly as for albumins, the lowest solubility was determined for GT; we noticed a decrease of up to 42% in ionic strength at 100 mM NaCl. For GC and Q samples, the application of higher concentrations of NaCl (100–500 mM) negatively affected solubility as compared to lower doses of NaCl (0–50 mM).

### 2.6. FT-IR Spectral Analysis

**Figure 9:**
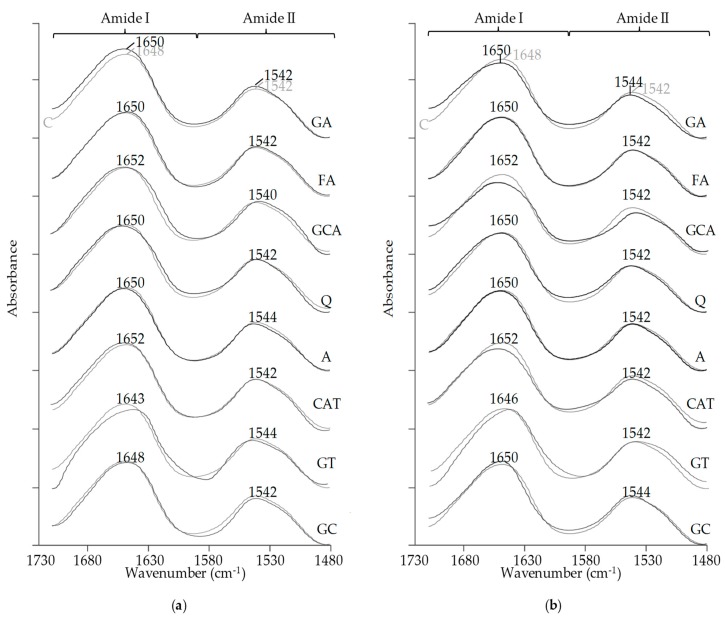
The effect of phenolic compounds on the FT-IR spectra absorbance at regions associated with amides I and II: (**a**) albumins; and (**b**) globulins. C–control sample (grey line), GA, FA, CGA, Q, A, CAT, GT, GC–protein samples after incubation with gallic acid, ferulic acid, chlorogenic acid, quercetin, apigenin, catechin, green tea, and green coffee extract, respectively.

The effect of phenolic compounds on the FT-IR absorbance spectra of globulins and albumins is depicted in Figure 9.

The FT-IR spectroscopic technique is commonly used for exploring the structural properties of proteins. Samples were monitored at 1700–1480 cm^−1^ regions which provide information concerning changes in the secondary structure of proteins. For all samples, we observed two typical peaks at ∼1650 cm^−1^ and ∼1540 cm^−1^ that were associated with amides I and II, respectively. The differences in the spectra between control samples and those treated by phenolic compounds were used as fingerprints of changes in the protein conformation, which is described in the discussion (Section 3.6). The results show that most of the phenolic compounds we applied had an effect on the FT-IR profiles of proteins as shown in Figure 9 (a, albumins and, b, globulins). The most significant changes in the peak symmetry of albumins were determined for GT and GA samples. In contrast, FA and A had only a slight effect on the FT-IR spectra of albumins. In case of globulins, changes in FT-IR profiles were more significant, and significant differences in absorbance spectra were observed for GT, CAT, CGA, and GA. Similarly as for albumins, the least significant effect was noticed for FA and A samples.

### 2.7. Binding Capacity of Proteins for Phenolic Compounds and Stability of Complexes

The results depicted in Table 1 show the binding capacity of proteins with regard to phenolic compounds. Among the “pure” phenolic compounds used in this experiment, the highest binding affinity to both albumins and globulins was observed for CGA—174.6 and 187.5 µmol/g, respectively. On the other hand, FA and A showed the least binding of all samples. FA was bound in amounts of 5.5 and 8.9 µmol/g and A at the level of 6.2 µmol/g by albumins and globulins, respectively.

Among the tested phenolics, GA and Q were bound in significantly higher amounts by albumins than by globulins; however, an inverse relationship was observed for CGA. In the case of plant extracts being a source of phenolics, six compounds for GT and seven for GC were monitored. For GT, they were (−)-gallocatechin (GCAT), (−)-epigallocatechin (EGCAT), (+)-catechin (CAT), (−)epicatechin (ECAT), (−)-epigallocatechin-3-gallate (EGCATG), and (−)-epicatechin gallate (ECATG); for GC, they were neochlorogenic acid (NCGA), CGA, cryptochlorogenic acid (CCGA), 3-feruloylquinic acid (FQA), 3,4-dicafyloqunic acid (3,4-DCQA), 3,5-dicafyloqunic acid (3,5-DCQA), and 4,5-dicafyloqunic acid (4,5-DCQA). Interestingly, the amount of main phenolic compounds that were bound from extracts (CAT for GT; CGA for GC) was significantly higher than that for “pure” phenolic compounds (CAT and GT). Additionally, catechin from GT and CGA from GC were bound in a significantly higher level by globulins than albumins. Furthermore, a significantly higher affinity to globulins was observed for GCAT and ECAT. Only ECATG were bound in statistically lower amounts. Similarly, in the case of GC extracts, most of their phenolics were characterized by a higher binding affinity to globulins than to albumins.

The effect of pH and ionic strength on the stability of protein–phenolic complexes is presented in the supplementary information (Table S1). Samples were analyzed at pH 2, 7, and 10 as well as at two ionic strengths (0 M NaCl and 1 M NaCl). At pH 7, pH 10, and ionic strength in native conditions (0 M NaCl) all tested phenolic compounds were not detected, thus these results were not presented in the Table S1. The results show that, at pH 7 and 10 and in the presence of 1 M NaCl, the albumin and globulin complexes with phenolic compounds were stable (phenolics were not detected). In general, at pH 2 and higher ionic strength (1 M NaCl), relatively slight amounts of phenolic compounds were released from proteins as compared to the bound fraction (Table 1). Only FA was released in higher amounts, which was a significant part of the amount dosed; however, it should be noted that this compound was bound at a relatively low quantity (Table 1). Among “pure” phenolic compounds, the highest amounts of released phenolics was determined for CAT. It was 6.4 and 7.1 µmol/g for albumins and 5.4 and 8.3 µmol/g for globulins, respectively, for pH 2 and at an ionic strength of 1 M NaCl. Relatively high amounts of compounds were released from GT and GC complexes, where the main compounds detected were CAT and CGA. For CAT (from GT), it was 8.3 and 4.3 µmol/g of albumins and 7.3 and 6.5 µmol/g of globulins; for CGA, it was 6.5 and 4.9 µmol/g of albumins and 7.2 and 3.7 µmol/g of globulins, respectively, at pH 2 and an ionic strength of 1 M NaCl. Additionally, as shown in Table S1, we observed statistically significant differences between their fractions in the stability of their complexes. For example, at pH 2, significantly higher amounts of CGA (“pure” compound) were released from globulins (4.7 µmol/g of protein) compared to that from albumins (3.6 µmol/g of protein). Conversely, albumin complexes with CGA were less stable than those with globulin when an ionic strength of 1 M NaCl was applied.

## 3. Discussion

### 3.1. UV-Vis Spectra of Protein–Phenolic Complexes

Phenolic compounds are well-known protective agents in plants against the effects of UV radiation. They absorb UV radiation and protect plants against its harmful effects, such as DNA damage. Furthermore, these compounds absorb light in the visible range, especially flavonoids which are well-known plant pigments [20].

The obtained results show that phenolic compounds significantly change the absorption properties of tested protein fractions, which are presented in Figure 1. The increase in overall UV-Vis absorption and in absorption at specific ranges are consequences of protein–phenolic interactions. Bonding with phenolics results in the increased absorption of complexes due to the ability of these compounds to absorb UV-Vis radiation. Similar results were obtained in previous studies on soy glycinin [18] and bovine serum albumin [21] treated with phenolic compounds. These studies show that the increase of the absorption of protein–phenolic complexes at specific wavelength ranges corresponds with the absorption properties of individual phenolic compounds. In our study, similar observations were recorded (Figure 1). For example, the maximum absorbance of CGA in the UV-Vis range is ~325 nm (results not published), which corresponds to the results obtained for protein samples where, at 325 nm, an increase of absorbance was observed for the CGA sample. Similar observations were made for Q, A, and CAT. Moreover, increasing absorbance at ~325 nm was observed for albumins and globulins and derivatized with GC phenolics, of which CGA was the main binding compound (Table 1).

### 3.2. Changes in the Content of Proteins, Free Amino Groups, Thiol Groups, and Tryptophan Residues Affected by Phenolic Compounds

Our investigations indicate that the interaction of phenolic compounds with white bean protein leads to a decrease in the amount of free amino, thiol, and tryptophan residues (Figure 2).

The covalent binding of phenolic compounds with amino acid-reactive groups cause blocking of the reaction sites for TNBS (2,4,6-Trinitrobenzenesulfonic acid) and DNTB (5,5′-Dithiobis(2-nitrobenzoic acid)); therefore, the abundance of free amino and thiol groups decreased after phenolic treatment of white bean proteins. Quenching of fluorescence is the result of a decrease of the yield of fluorescence from a fluorophore (tryptophan residue) that is induced by interactions with the quencher molecule (in this case, with a phenolic compound) [22].

Similarly as some previous studies, the highest reactivity against free amino groups, thiol groups, and tryptophan residues was observed for such substances as GA, CGA, and quercetin [18,23,24].

The reaction of phenolics with the reactive sites of proteins such as free amino groups, thiol groups, and tryptophan residues occurs after their oxidative conversion to reactive quinones. Polyphenols undergo oxidation relatively easily, which is attributed to their structure—the presence of hydroxyl groups (-OH) groups located on the aromatic rings. Phenolic compounds may be oxidized in enzymatic processes (e.g., reactions catalyzed by polyphone oxidase) as well as non-enzymatically. Non-enzymatic oxidation occurred under relatively mild conditions during food processing; however, high temperature, alkaline environment, and the presence of oxygen and pro-oxidants enhances this process [19,21,25,26].

The highly reactive quinones easily undergo attack as electrophiles by nucleophilic amino acid side chains such as in lysine, cysteine, methionine, and tryptophan in protein molecules which causes their binding by covalent bonds. For phenolics with higher amounts of hydroxyl groups, the next reactions may occur to lead to protein aggregation [21,24]. Additionally, a single phenolic molecule (with at least two hydroxyl groups) are able to link different fragments of the polypeptide chain of the same protein molecule. Thus, reaction mechanisms depend on the phenolic structure and molecular characteristics. Examples of the reaction of phenolic compounds with amino acid side chains have been widely discussed in some previous works [19,21,25].

Furthermore, it may be speculated that the amount of free groups of amino acids may be affected by the indirect action (secondary effect) of phenolics. As observed (Figure 9) and described, phenolic compounds are able to affect protein conformation (secondary structures) and, thus, structural changes (which may be also caused by non-covalent interactions) may limit or induce the availability of amino acid residues for complexing with other phenolic molecules. Additionally, it should be mentioned that the complex spatial structure of proteins may limit the availability of reactive sites for phenolic molecules; thus, only a part of all free amino groups were blocked (Figure 2).

Our results show that the affinity of phenolics to side chains of amino acids is dependent on the analyzed protein fraction, which indicates that factors other than phenolic-related factors affect the interactions with amino acid-reactive groups, for example, the amount, type, and accessibility of reactive sites in protein molecules.

### 3.3. Size-Exclusion High-Performance Liquid Chromatography

SE-HPLC is a method that is commonly used for protein separation and analysis of their molecular size distribution. In this study, SE-HPLC was applied to visualize the complexation of proteins and phenolics.

Similar as in our study, significant effects of phenolic compounds on SE-HPLC profiles of proteins were noticed in the study of Hanato et al. [27] where bovine serum albumin was treated with (−)-epigallocatechin gallate, oenothein B, and cornusiin A, among others. Additionally, size-exclusion chromatography was used for the analysis of protein–phenolic complexes formed during honey storage [28].

Compared with electrophoresis, separation takes place in milder chemical behavior; thus, it may allow the demonstration of the occurrence of more subtle interactions such as those formed by non-covalent bonds. Conversely, the usefulness of this method is limited to analysis of proteins and protein–phenolic complexes which are soluble under the separation conditions [27].

It may be speculated that changes in the chromatographic profiles of white bean proteins after mixing with phenolic compounds is a result of both intra-and intermolecular interactions. Shifting some peaks to a lower retention time is associated with an increase of the molecular size of protein subfractions. The appearance of new peaks corresponding to higher molecular sizes and decreased peak size or the disappearance of some peaks may be caused by the involvement of proteins in the formation of intermolecular complexes (rearrangement of protein subfractions affected by cross-linking). Furthermore, an increase of some peak sizes may be an effect of the interaction of protein molecules with phenolic compounds, and an increasing in the absorbance of complexes (e.g., intermolecular complexes) is able to increase the absorbance of protein at a detection wavelength of 280 nm (as it was described in Section 2.1).

### 3.4. Polyacrylamide Gel Electrophoresis

Polyacrylamide gel electrophoresis of white bean proteins shows significant changes in the profiles of albumins and globulins affected by most of the phenolic compounds that are used. Similar effects were described for whey proteins [23], myoglobin [24], lysozyme [29], and soy proteins [18] treated with various phenolic compounds. Additionally, in some studies, an insignificant influence on the electrophoretic profiles for FA and apigenin was observed. Nevertheless, the strong influences of CGA, GA, and Q on electrophoretic patterns were observed [18,23,24]. In the following studies, interactions of these phenolic compounds with proteins resulted in the formation of dimers and higher molecular complexes [18,23,24].

Furthermore, the presence (or increase of abundance) of the cross-linked protein complexes which remained at the top of the stacking gels or in the range of higher molecular weights (e.g., fraction >250 kDa) were noticed in our study (Figure 5 and Figure 6). Conversely, a decrease in the abundance of some protein subfractions (band disappearance, decrease in band intensity; Figure 5 and Figure 6) indicated that these fractions participate in the formation of cross-linked protein–phenolic complexes, as was also described by Rawel et al. [18]. Intermolecular protein–phenolic complexes were found even when denaturing and reducing agents—SDS and β-mercaptoethanol, respectively (Figure 5)—were applied. SDS breaks the hydrophobic interactions and hydrogen bonds, and β-mercaptoethanol is used to break disulfide bonds [30]. Therefore, it can be assumed that the resultant protein–phenolic cross-links were stabilized by covalent bonds. Conversely, it can be supposed that changes in molecular composition determined by Native-PAGE may be caused by non-covalent interactions, because separation was carried out without denaturing and reducing agents.

Furthermore, it may be speculated that changes in electrophoretic patterns may be a consequence of the modification of protein charge, which affects the molecule’s mobility during the separation process. Previous studies on isoelectric focusing of protein molecules show that phenolic substances are able to shift their isoelectric point [18,23,29]. Authors reported that this effect may be caused by the attachment of phenolics to charged groups of proteins such as free amino groups (blocking of positively charged groups, increase in the negative charge of the molecules) and/or due to the introduction the negatively charged carboxylic groups in case of phenolic acids [18,23,29]. Probably, a similar mechanism could be involved in SDS-PAGE, where phenolic–protein interactions may affect the binding of SDS and protein and, finally, change the charge of the separating molecules. However, to our knowledge, there is no evidence in the literature to confirm this speculation.

### 3.5. Effect of pH and Ionic Strength on the Solubility of Protein–Phenolics Complexes

Solubility is one of the main physicochemical parameter that characterizes the functional properties of food proteins (e.g., foaming capacity and foam stability, emulsion capacity, stability) [31]. Furthermore, it is an important technological parameter that influences the protein isolation efficiency. Besides this, as described in work of Carbonaro et al., solubility determines the nutritional properties of proteins, such as susceptibility to digestion, which was described for legume proteins [32].

Solubility is affected by extrinsic factors, including pH, ionic strength, temperature, various solvent additives, and intrinsic factors, that are determined by protein structure and amino acid composition such as surface net charge and hydrophobicity [33].

The results obtained in our study show that most phenolic compounds were effective modifiers of the solubility of white bean proteins that were affected by pH (Figure 7) and ionic strength (Figure 8). However, the intensity of this effect is dependent on the type of phenolic substances that are applied and the type of protein fraction that was analyzed. In general, phenolics had a negative effect on albumin solubility; however, an increase in globulin solubility was affected by pH, although strength results were diversified in the case of ionic. The following observations indicate the important role of the type of protein fraction and, consequently, their physicochemical and molecular properties on the solubility profile after phenolic treatment.

A similar differentiation in solubility of soy proteins (soy trypsin inhibitor, soy glycine) caused by phenolic compounds was noticed in the study of Rawel et al. [18]. Significant effects (both positive and negative) of phenolics on the solubility and/or extractability of various proteins were observed in other studies [21,23,24,34,35]. Phenolics can induce conformational changes in protein structure, leading to the exposure of some hydrophilic or hydrophobic regions that were previously buried and, thereby, modify the conjugation process between molecules. Changes in secondary structure were also described in our study (Figure 9). Moreover, blocking of hydrophobic and hydrophilic residues of amino acids (e.g., tryptophan and lysine ε-amino group, respectively) as well as incorporation of polar carboxylic groups from phenolic acids are involved in the solubility of proteins [18]. Increased protein hydrophobicity can cause the association of molecules by hydrophobic interactions as well as their precipitation, which conversely decreases the hydrophobicity and positively influences their hydration and resultant solubility [21,33]. Moreover, protein precipitation may be caused by covalent cross-linking of protein molecules by phenolic substances as described in Section 3.2.

### 3.6. FT-IR Spectral Analysis

Previous investigations have shown that FT-IR spectroscopy is a useful tool for analysis of changes in molecular conformation of proteins affected by protein–phenolic interactions [36,37,38]. Functional groups of proteins responsible for their conformation exhibit the absorption of infrared radiation at characteristic frequencies; therefore, differences in the FT-IR spectra are fingerprints of their conformational changes in the secondary structure [39,40].

As it was described in this study, we analyzed a region over the range 1700–1480 cm^−1^. This region is attributed to signals of amides I and II. In amide I (∼1650 cm^−1^), the absorption of IR radiation causes a stretching vibration of mainly the C=O bond (80% C=O stretch, 10% C―N stretch, 10% other), whereas in amide II (∼1550 cm^−1^), it mainly causes a bending vibration of the N–H bond (60% N―H bend, 30% C―N stretch, and 10%, C―C stretch) [36,39,40,41].

Barth reported that amide I and amide II vibration modes strongly depend on the secondary structure of proteins, and they are hardly affected by the nature of their side chains; thus, they are suitable for analysis of secondary structure; however, the correlation between structure and frequency is more straightforward for the amide I modes [39].

It is well-known that the secondary structure of proteins is stabilized by hydrogen bonds between functional groups of amino acids, including C=O and N–H. The rearrangement in the hydrogen-bonding network of the protein molecule cause changes in amide vibrational modes, which is reflected in the FT-IR spectra and structural properties of protein molecules [36,39].

The interactions of phenolic compounds with amino acid residues (Figure 2) are able to reorganize the hydrogen-bonding network and, finally, the protein structure. Similarly as in this study, a significant influence of phenolic compounds on the secondary structure of proteins was also noticed previously. For example, tea polyphenols (catechin, epicatechin, epigallocatechin, and epigallocatechin gallate) altered the structure of caseins with a reduction of the α-helix and β-sheet and an increase of random coil and turn structure [22]. In another study, where the effect of removal phenolics on the structure of sunflower proteins was examined, the presence of phenolic substances increased in the unordered structure at the expense of the ordered structures [36].

### 3.7. Binding Capacity of Protein for Phenolic Compounds and Stability of Complexes

The results presented in Table 1 show that the binding capacity of phenolic compounds to proteins were diversified by the type of phenolic compound and protein fraction used in the experiment. Most phenolic compounds were bound at relatively high amounts, which influenced the other measured parameters. On the other hand, FA and A were bound at significantly lower levels, and these compounds had only a slight effect on the physicochemical properties of proteins. On the other hand, the amount of bound phenolic compounds was not only a factor that influenced the characteristics of protein derivatives. In most cases, a unique combination of phenolic compounds/extracts and protein fractions differentiated the affinity properties of protein–phenolic complexes and resulted in their physicochemical properties.

Surprisingly, CGA and CAT from extracts (GC and GT samples) were bound in larger quantities than from solutions of pure compound. It seems likely that that these effects may be caused by co-introduced compounds that lead to conformational changes in protein molecular structure and expose new binding sites for CGA and CAT. Furthermore, it was not reflected in the changes of physiochemical properties which, in some cases, were more noticeable when “pure” compounds were applied.

The obtained protein–phenolic complexes are generally stable under the applied pH and ionic strength conditions (Table S1). Slight amounts of desorbed phenolics were detected at pH 3 in 1 M NaCl. This may indicate that the formed complexes were stabilized mainly by irreversible covalent bonds, and only a small amount of dosed compounds were bound by other reversible interactions. Furthermore, it may be speculated that proteins are able to entrap phenolic compounds in their spatial structure, and considerable changes of pH and ionic strength cause conformation and molecular rearrangement leading to the release of phenolics.

## 4. Materials and Methods

### 4.1. Chemicals

Gallic acid (GA), ferrulic acid (FA), chlorogenic acid (CGA), quercetin (Q), (+)-catechin (CAT), (−)-gallocatechin (GCAT), (−)-epigallocatechin (EGCAT), (−)epicatechin (ECAT) (−)-epigallocatechin-3-gallate (EGCATG), (−)-epicatechin gallate (ECATG), neochlorogenic acid (NCGA), cryptochlorogenic acid (CCGA), 3-feruloylquinic acid (FQA), 3,4-dicafyloqunic acid (3,4-DCQA), 3,5-dicafyloqunic acid (3,5-DCQA) 4,5-dicafyloqunic acid (4,5-DCQA), Bradford reagent, 2,4,6-trinitrobenzenesulfonic acid (TNBS) and 5,5′-Dithiobis(2-nitrobenzoic acid (DTNB) were purchased in Sigma–Aldrich company (St. Louis, MO, USA). Apigenin (A) was purchased from the Roth company (Karlsruhe, Germany). All others chemicals were of analytical grade.

### 4.2. Plant Materials

Commercially available white kidney beans (*Phaseolus vulgaris* (L.) var. Jas Karlowy), green coffee beans (*Coffea arabica* L.) and green leaf tea (*Camellia sinensis* (L.) Kuntze) were purchased from a local store.

### 4.3. Extraction of Protein Fractions

White kidney beans were powdered using a laboratory mill and sieved (sieve with 0.5 mm square holes). White bean flour (WBF) was defeated using n-hexane (flour/n-hexane ratio; 1/30; *w*/*v*) with continuous stirring for 4 h at room temperature. The suspension was left to decant for 1 h and supernatant was removed. Then, the pellet was air-dried for 16 h at 40 °C. Albumin and globulin fractions were separated based on solubility criteria; according to improved procedure of Ribeiro et al. with some modifications [42].

#### 4.3.1. Isolation of Albumins

To obtain the albumin fraction, the defatted WBF was mixed with 10 mM CaCl_2_ and 10 mM MgCl_2_ in distilled water, and pH was adjusted to 8 with 2 M sodium hydroxide (NaOH). The flour/extraction solution ratio was 1/30 (*w*/*v*). The suspension was continuously stirred for 4 h at 4 °C. Then, the insoluble residues were removed by centrifugation at 9000× *g* at 4 °C for 30 min. The supernatant containing the albumin fraction was transferred into the cellulose dialysis tube (Sigma–Aldrich), and the proteins were dialyzed for 24 h at 4 °C against distilled water and then lyophilized. The lyophilized albumins were stored in closed plastic containers in the dark at −40 °C until analysis.

#### 4.3.2. Isolation of Globulins

For globulin extraction, the insoluble residues obtained after albumin isolation were resuspended in 100 mM Tris-HCl buffer (pH 7.5) containing 10% (*w*/*v*) NaCl, 10 mM EDTA, and 10 mM EGTA. The flour/extraction solution ratio was 1/30 (*w*/*v*). The suspension was continuously stirred for 4 h at 4 °C. Then, the insoluble residues were removed by centrifugation at 9000× *g* at 4 °C for 30 min. The globulins in the supernatant were precipitated with ammonium sulfate (561 g/L) and centrifuged at 9000× *g* at 4 °C for 30 min. The globulins were resuspended in 50 mM Tris-HCl buffer (pH 7.5), transferred into the cellulose dialysis tube (Sigma–Aldrich, St. Louis, MO, USA), and the proteins were dialyzed for 24 h against distilled water and then lyophilized. The lyophilized globulins were stored in closed plastic containers in the dark at −40 °C until further analysis.

### 4.4. Extraction of Phenolic Compounds from Plant Materials

The green coffee and green tea extracts were prepared as follows: green coffee beans and green tea leaves was powdered using a laboratory mill and sieved (sieve with 0.35 mm square holes). Then, powdered green coffee (8 g) and green tea (4.8 g) were brewed with 400 mL of boiled distilled water, allowed to stand until cool at room temperature for 1 h and filtered through PTFE membrane filter (pore size 0.45 μm).

### 4.5. Incubation of Proteins with Phenolic Compounds

The proteins were incubated with phenolic compounds according to the procedure described by Rawel et al. [23] but with slight modifications. The protein–phenolic solutions were prepared by mixing ethanolic solution of pure phenolic compounds (GA, FA, CGA, Q, A, and CAT—0.3 mmol/5 mL) with 1 g of albumins or globulins suspended in 95 mL distilled water. The pH of reaction mixtures was adjusted to 9, and the samples were incubated for 24 h at 4 °C with continuous mixing. After incubation, the samples were transferred into the cellulose dialysis tube (Sigma–Aldrich), dialyzed for 24 h at 4 °C against distilled water, and lyophilized.

The incubation of proteins with phenolics from plant materials was conducted as follows: 1 g albumin and globulin were suspended in 50 mL distilled water, and the solutions were transferred into a dialysis tube and placed in plastic containers filled with 250 mL GT or GC extracts and the pH was adjusted to 9 with NaOH. After incubation, the protein samples were transferred to another container and dialyzed for 24 h at 4 °C against distilled water. Control samples were prepared under the same conditions but without the addition of phenolics.

### 4.6. UV-Vis Spectra of Protein–Phenolic Complexes

The UV-Vis absorption were determined according to procedure Rawel et al. [18] with slight modifications. Protein samples were dissolved in 100 mM sodium phosphate buffer pH 7 (0.1 mg of protein/mL) and 250 µL of each sample was transferred into UV-Star 96-well microplate (Greiner Bio-One, Frickenhausen, Germany). The UV-Vis spectra were measured at the range of 200 to 600 at 20 °C using EPOCH 2 microplate reader (BioTek, Winooski, VT, USA).

### 4.7. Changes in Content of Proteins Free Amino Groups, Thiol Groups, and Tryptophan Residues Affected by Phenolic Compounds

#### 4.7.1. Effect of Phenolic Compounds on The Content of Free Amino Groups

The effect of phenolic compounds on the content of free amino groups was determined using the TNBS method [43] with modifications. Briefly, 10 µL of protein solutions (10 mg protein/mL 1% SDS) were mixed with 30 µL 1% SDS and 40 µL 0.1 TNBS in 1% SDS, and incubated in the dark at 50 °C. After 60 min, 80 µL 1 M HCl was added to stop the reaction, and the mixture was cooled to room temperature for 10 min. Absorbance was measured at 340 nm using the EPOCH 2 microplate reader (BioTek, VT, USA). For each analyzed sample, two blank samples—B1 and B2—were prepared under the same conditions. B1 was made by replacing solutions of proteins with 1% SDS, and B2 was made by replacing TNBS solution with 1% SDS, respectively. The relative content of free amino groups was calculated as follows:

(1) $${\mathrm{RC}}_{A}=\frac{A_{P}}{A_{C}}\times 100\%$$

where RC_A_—relative content of free amino groups in the sample treated by phenolics (%); A_P_—content of free amino groups in the sample treated by phenolics (absorbance at 340 nm) and A_C_—content of free amino groups in the control sample (absorbance at 340 nm)

#### 4.7.2. Effect of Phenolic Compounds on the Content of Free Thiol Groups

The effect of phenolic compounds on the content of free thiol groups was determined using the TNBS method [44] with modifications. Briefly, 20 µL of protein solutions (10 mg protein/mL 1% SDS) were mixed with 20 µL 0.1% DTNB in 1% SDS and 220 µL 1% SDS. Samples were incubated at room temperature for 30 min and absorbance was measured at 412 nm using EPOCH 2 microplate reader (BioTek, Winooski, VT, USA). For each analyzed sample, two blank samples—B1 and B2—were prepared under the same conditions. B1 was made by replacing solutions of proteins with 1% SDS, and B2 was made by replacing TNBS solution with 1% SDS, respectively. The relative content of free amino groups was calculated as follows:

(2) $${\mathrm{RC}}_{T}=\frac{T_{P}}{T_{C}}\times 100\%$$

where RC_T_—relative content of free thiol groups in the sample treated by phenolics (%); T_P_—content of free thiol groups in the sample treated by phenolics (absorbance at 412 nm) and T_C_—content of free thiol groups in the control sample (absorbance at 412 nm)

#### 4.7.3. Effect of Phenolic Compounds on the Free Tryptophan Residues

The tryptophan fluorescence quenching experiment was conducted according to the procedure described by Rawel et al. but with some modifications [45]. Protein samples were dissolved in 100 mM sodium phosphate buffer and centrifuged (9000× *g*) at 20 °C for 10 min. Next, the protein solution was transferred into a quartz quvette. Samples were excited at 280 nm (slit 10 nm) and emission was recorded at 340 nm (slit 10 nm) using a Quantech Digital Filter Fluorometer (Thermo Scientific, Waltham, MA, USA) against blank samples. The relative content of free tryptophan residues was calculated as follows:

(3) $${\mathrm{RC}}_{\mathrm{TRP}}=\frac{{\mathrm{TRP}}_{P}}{{\mathrm{TRP}}_{C}}\times 100\%$$

where RC_TRP_—relative content of free tryptophan residues in the sample treated by phenolics (%); TRP_P_—content of free tryptophan residues in the sample treated by phenolics (fluorescence 340 nm) and TRP_C_—content of free tryptophan residues in the control sample (fluorescence 340 nm)

### 4.8. Size-Exclusion High-Performance Liquid Chromatography

Proteins were analyzed by SE-HPLC using a Varian ProStar HPLC System separation module (Varian, Palo Alto, CA, USA) equipped with a column (COSMOSIL 5-Diol-300-II Packed Column 7.5 mm ID × 600 mm, Nacalai Tesque, Kyoto, Japan) and a ProStar diode array detector (DAD). The column thermostat was set at 30 °C. The protein water solutions (20 mg/mL in phosphate-buffered saline [PBS]) were loaded on the column in samples of 50 µL, and proteins were eluted using a PBS buffer (pH 7.4). The flow rate was 1 mL/min. Ultraviolet detection was undertaken at a wavelength of 280 nm.

### 4.9. Polyacrylamide Gel Electrophoresis

#### 4.9.1. SDS-PAGE

Proteins were analyzed by SDS-PAGE according to the modified Laemmli procedure [46]. Samples were separated in 4% (*w*/*v*) stacking gel (126 mM Tris-HCl, pH 6.8; 0.001% SDS; 0.001% TEMED; 0.005% APS) and 12% (*w*/*v*) resolving acrylamide gel (375 mM Tris-HCl, pH 8.8; 0.001% SDS; 0.0005% TEMED; 0.0005% APS). Samples for electrophoresis were prepared as follows: 10 mg of proteins were mixed with 250 µL distilled water and 250 µL 2× Laemmli sample buffer (65.8 mM Tris-HCl pH 6.8; 26.3% (*w*/*v*) glycerol; 2.1% SDS; 0.01% bromophenol blue; 355 mM β-mercaptoethanol; BioRad, Hercules, CA, USA). Samples were boiled for 5 min, and 20 µL of protein solution was loaded into each lane. The electrophoresis was carried out at a constant voltage of 20V during stacking gel and 100 V during resolving gel at 4 °C using a Mini-PROTEAN Tetra Cell (BioRad). Gels, after electrophoresis, were washed in solution of 10% acetic acid in 50% methanol for 20 min, then in distilled water for 1 min (2 times), and stained with 0.1% Coomassie Brilliant Blue in 10% acetic acid in 40% methanol, and destained in 10% acetic acid in 40% methanol. The molecular mass of protein was evaluated using BioRad Precision Plus Protein Standards in the range 10–250 kDa. After electrophoresis, protein patterns were analyzed with GelAnalyser software.

#### 4.9.2. Native-PAGE

Protein samples were analyzed by Native-PAGE according to the modified Wittig et al. procedure [47]. Samples were separated in 4% (*w*/*v*) stacking gel (126 mM Tris-HCl, pH 6.8; 0.001% TEMED; 0.005% APS) and 10% (*w*/*v*) resolving acrylamide gel (375 mM Tris-HCl, pH 8.8; 0.001% SDS; 0.0005% TEMED; 0.0005% APS). Samples for electrophoresis were prepared as follows: 10 mg of proteins were mixed with 250 µL distilled water and 250 µL 2× sample buffer (65.8 mM Tris-HCl pH 6.8; 26.3% (*w*/*v*) glycerol; 0.01% bromophenol blue; BioRad) and 20 µL of protein solution was loaded into each lane. The electrophoresis was carried out at a constant voltage of 20 V during stacking gel and 100 V during resolving gel at 4 °C using a Mini-PROTEAN Tetra Cell (BioRad). Gels, after electrophoresis, were washed in solution of 10% acetic acid in 50% methanol for 20 min, then in distilled water for 1 min (2 times), and stained with 0.1% Coomassie Brilliant Blue in 10% acetic acid in 40% methanol, and destained in 10% acetic acid in 40% methanol. After electrophoresis, protein patterns were analyzed with GelAnalyser software.

### 4.10. Effect of pH and Ionic Strength on The Solubility of Protein–Phenolics Complexes

#### 4.10.1. Effect of pH

Protein samples (10 mg) were mixed with 200 µL wide range buffer (pH 2–12) prepared according to Carmody’s method [48]. The mixture was incubated for 30 min at 20 °C with continuous shaking. Thereafter, samples were centrifuged (10,000× *g*) at 4 °C for 10 min. The supernatant (5 µL) was mixed with 250 µL of Bradford Reagent (Sigma–Aldrich) and incubated for 15 min at 20 °C with continuous shaking. The absorbance at 595 nm was measured in EPOCH 2 microplate reader (BioTek). For each sample, under the same conditions, blank samples—B1 and B2—were prepared, where B1 was made by replacing solutions of protein with appropriate buffer, and B2 was made by replacing Bradford reagent with methanol. The relative solubility of protein was expressed as a percentage difference between the absorbance of the most soluble control sample and the analyzed sample. Obtained results show that the highest solubility of control samples for albumins and globulins was in pH 11 (solubility = 100%). Thus, the relative solubility of treated by phenolic compounds were calculated as follows:

(4) $${\mathrm{RS}}_{\mathrm{pH}}=\frac{\mathrm{AP}}{{\mathrm{AC}}_{\mathrm{pH}\;11}}\times 100\%$$

where RS_pH_—relative solubility of protein affected by pH (%); AP—absorbance of sample treated by phenolics and AC_pH 11_—absorbance of control sample in pH 11

#### 4.10.2. Effect of Ionic Strength

Protein samples (5 mg) were mixed with 1 mL of 0, 50, 100, 250 and 500 mM solutions of NaCl in distilled water. The mixtures were incubated for 30 min at 20 °C with continuous shaking. Thereafter, samples were centrifuged (10,000× *g*) at 4 °C for 10 min. The supernatant (5 µL) was mixed with 250 µL of Bradford Reagent (Sigma–Aldrich) and incubated for 15 min at 20 °C with continuous shaking. The absorbance at 595 nm was measured in EPOCH 2 microplate reader (BioTek). For each sample, under the same conditions, blank samples—B1 and B2—were prepared, where B1 was made by replacing solutions of protein with appropriate NaCl solution, and B2 was made by replacing Bradford reagent with methanol. The relative solubility of protein was expressed as a percentage difference between the absorbance of control sample in distilled water (0 M of NaCl) and analyzed sample. The relative solubility of samples treated by phenolic compounds were calculated as follows:

(5) $${\mathrm{RS}}_{\mathrm{IS}}=\frac{\mathrm{AP}}{{\mathrm{AC}}_{\mathrm{OM}}}\times 100\%$$

where S_IS_—relative solubility of protein affected by ionic strenght (%); AP—absorbance of sample treated by phenolics and AC_0M_—absorbance of control sample in 0 M NaCl

### 4.11. FT-IR Spectral Analysis

A Direct Detect Infrared Spectrometer (Merck Millipore, Darmstadt, Germany) was used to obtain spectrograms of protein samples. Protein samples of 5 mg were mixed with 2.5 mL PBS buffer (pH 7.4) and 2 µL of sample was transferred into a hydrophilic polytetrafluoroethylene (PTFE) membrane engineered for sample application and retention (Merck Millipore). In a blank sample, 2 µL PBS buffer was applied to the membrane. The FT-IR spectra in the region of 1700–1480 cm^−1^ were collected with a 2 cm^−1^ spectral resolution.

### 4.12. Binding Capacity of Protein for Phenolic Compounds and Stability of Complexes

#### 4.12.1. Binding Capacity of Protein for Phenolic Compounds

After incubation of phenolic compounds with proteins (procedure described in Section 4.5), a sample of the protein–phenolic mixture (5 mL) was transferred into the dialysis cell (Amicon Stirred Cell 8010, Merck Millipore, Darmstadt, Germany) and free phenolics were separated by diafiltration using a 10-kDa regenerated cellulose membrane (Merck Millipore). During diafiltration, the samples were washed three times with distilled water (5 mL). The content of phenolics was determined by HPLC analysis, as described in Section 4.13. The amount of bound phenolics was calculated on the basis of the difference in the amount of phenolics dosed and free phenolics after incubation with proteins.

#### 4.12.2. Effect of pH on the Desorption Stability of Protein–Phenolic Complexes

Protein samples (50 mg) were mixed with 4 mL distilled water, and pH was adjusted with 1 M HCl or 1 M NaOH to 2, 7, and 10. The mixtures were made up to 5 mL with distilled water and incubated for 1 h at 20 °C with continuous shaking. Then, samples were transferred into dialysis cells (Amicon Stirred Cell 8010, Merck Millipore) and phenolic compounds were separated by diafiltration using a 10-kDa regenerated cellulose membrane (Merck Millipore). During diafiltration, the samples were washed three times with distilled water (1 mL) at the appropriate pH. The content of phenolic compounds was determined by HPLC analysis (Section 4.13.)

#### 4.12.3. Effect of Ionic Strength on the Desorption Stability of Protein–Phenolic Complexes

Protein samples (50 mg) were mixed with 5 mL distilled water (0 M NaCl) or 1 M NaCl solution, and incubated for 1h at 20 °C with continuously shaking. Then, samples were transferred into dialysis cells (Amicon Stirred Cell 8010, Merck Millipore) and phenolic compounds were separated by diafiltration using a 10-kDa regenerated cellulose membrane (Merck Millipore). During diafiltration, the samples were washed three times with 1 mL of distilled water or 1 M NaCl. The content of phenolic compounds was determined by HPLC analysis (Section 4.13.).

### 4.13. Determination of Phenolic Contents by HPLC

Samples were analyzed with a Varian ProStar HPLC system equipped with a COSMOSIL 5C18-MS-II Packed Column 25 mm × 4.6 mm and a ProStar DAD detector. The column thermostat was set at 30 °C. The mobile phase was 0.1% formic acid in water (solvent A) and 0.1% formic acid in acetonitrile (solvent B) at a flow rate of 1 mL/min. Pure phenolic compounds were determined using elution mode as follows: 0–5 min isocratic elution 5% B, 5–20 min linear gradient elution from 5% B to 100% B, 20–25 min isocratic elution 100% B, 25–30 min linear gradient elution from 100% B to 5% B. For determination of phenolic compounds from green coffee analytes were eluted with 5% B for 5 min, followed by linear gradient to 40% B from 5 min to 40 min, 40% B was maintained constant up to 45 min and linear gradient elution from 40% B to 5% B for 5 min was used. In case of green tea samples elution conditions were 5% B for 5 min, a linear gradient from 5% B to 40% B (5–40 min), an isocratic elution 40% B (40–45 min) and a linear gradient from 40% B to 5% B (45–50 min). Gallic acid was detected at 270 nm, ferulic and chlorogenic acid at 325 nm, quercetin and apigenin at 350 nm, and catechin at 280 nm. For green tea (GT) and green coffee (GC) extracts phenolics detection was performed at 270 nm. For GT samples, quantity of GCAT, EGCAT, CAT, ECAT, EGCATG, and ECATG were determined. For GC quantity of NCGA, CGA, CCGA, FQA, 3,4-DCQA, 3,5-DCQA, and 4,5-DCQA were analyzed. Phenolics for plant materials were chosen based on literature reports and availability of commercial standards [49,50,51]. Quantitative analysis was carried out with the external standard calculation, using calibration curves.

### 4.14. Statistical Analysis

All experimental results represent the means values ± SD (standard deviation) for three parallel measurements. One-way analysis of variance, post hoc and Tukey’s tests were used to compare groups. α values = 0.05 were regarded as statistically significant.

## 5. Conclusions

So far, investigations were mainly focused on the protein–phenolic interactions in simple models, where single proteins and chemically pure phenolic compounds were applied. In this study more complicated systems were used to show the probable effects in food system, where multidirectional interactions are possible. Furthermore, plant derived proteins were subjected to analysis to show the relationships with main nutrients of staple foods. The results of our research indicate that phenolic compounds are able to complex with white bean albumins and globulins. Most of the analyzed phenolic compounds—both chemically “pure” and from plant extracts—had a significant influence on the physicochemical properties of protein fractions such as absorption of light in the UV-Vis range; amount of free amino groups, thiol groups, and tryptophan residues; chromatographic (SE-HPLC) and electrophoretic profiles (SDS-PAGE, Native-PAGE); as well as proteins solubility affected by pH and ionic strength. The analysis of FT-IR spectra at regions attributed to amides I and II demonstrated that most of the phenolic compounds induced conformational changes in the secondary structure of proteins. Additionally, to our knowledge, for the first time the binding capacity of proteins to phenolic compounds and stability of complexes at different pH and ionic strength were determined. Results show that the complexes were generally stable under the applied conditions, thus they were mainly stabilized by irreversible interactions. However, presence of reversible interactions were also found simultaneously. The least impact on the physicochemical properties was observed for FA and A, and these compounds were also bound at the least amount as compared to other compounds. Besides the amount of bound substances, the measured physicochemical parameters were differentiated by the type of phenolic compounds and protein fraction. Furthermore, it may be supposed that changes in properties of complexes reflect in the biological nature of proteins, such as their susceptibility to digestion and the bioavailability of amino acids. Conversely, irreversible binding of phenolic compounds may limit their absorption and ability to exert pro-health activity. Furthermore, the interactions of phenolics with food proteins seem to be an important factor influencing on the functional quality of phenolics and protein rich foods, and should be taken into account during the development of functional products. However, due to the structural complexity of proteins, the diversity of phenolic compounds in nature, the multitude of factors affecting their interactions, and the limitations of analytical methods, such studies pose a great and long-term challenge for the domain of food science.

## Figures and Tables

**Table 1:** Binding capacity of proteins for phenolic compounds.

Sample			Albumins	Globulins
			Phenolics Bound µmol/g Protein ± SD	
Phenolics		GA	172.2 ^Aa^ ± 5.32	157.4 ^Bc^ ± 4.02
		FA	5.5 ^Ac^ ± 2.15	6.2 ^Ae^ ± 2.32
		CGA	174.6 ^Ba^ ± 5.12	187.5 ^Aa^ ± 6.50
		Q	149.4 ^Ab^ ± 6.95	131.4 ^Bc^ ± 7.72
		A	8.9 ^Ac^ ± 2.24	7.1 ^Ad^ ± 2.22
		CAT	157.3 ^Ab^ ± 6.84	165.3 ^Ab^ ± 6.12
Extracts	GT	GCAT	5.2 ^Bf^ ± 0.82	11.3 ^Ad^ ± 0.61
		EGCAT	21.0 ^Ac^ ± 1.09	19.3 ^Ac^ ± 1.39
		CAT	174.3 ^Ba^ ± 2.25	184.6 ^Aa^ ± 4.37
		ECAT	8.6 ^Be^ ± 0.23	10.0 ^Ae^ ± 0.65
		EGCATG	89.3 ^Ab^ ± 1.09	86.2 ^Ab^ ± 2.97
		ECATG	12.1 ^Ad^ ± 0.54	8.5 ^Bf^ ± 0.63
	GC	NCGA	7.1 ^Bd^ ± 0.33	11.7 ^Ac^ ± 0.55
		CGA	212.9 ^Ba^ ± 8.12	231.5 ^Aa^ ± 7.11
		CCGA	10.9 ^Bb^ ± 1.18	18.5 ^Ab^ ± 2.72
		FQA	10.8 ^Bb^ ± 0.42	3.3 ^Af^ ± 0.43
		3,4-DCQA	2.9 ^B^ ± 0.30	3.8 ^Aef^ ± 0.59
		3,5-DCQA	8.9 ^Ac^ ± 0.61	9.7 ^Ad^ ± 1.71
		4,5-DCQA	3.9 ^Be^ ± 0.48	4.1 ^Ae^ ± 0.33

Means (± SD, *n* = 3) with different lowercase letters superscript within a same column for phenolics, GT extracts, and GC extracts are significantly different (α = 0.05). Means with different capital letters superscript within a same row for protein fractions are significantly different (α = 0.05). GA, FA, CGA, Q, A, CAT–gallic acid, ferulic acid, chlorogenic acid, quercetin, apigenin, catechin, respectively. Green tea (GT) compounds: GCAT ((−)-gallocatechin), EGCAT ((−)-epigallocatechin), CAT ((+)-catechin), ECAT ((−)epicatechin), EGCATG ((−)-epigallocatechin-3-gallate), and (−)-epicatechin gallate (ECATG); Green coffee (GC) compounds: NCGA (neochlorogenic acid), CGA (chlorogenic acid), CCGA (cryptochlorogenic acid), FQA (3-feruloylquinic acid), 3,4-DCQA (3,4-dicafyloqunic acid), 3,5-DCQA (3,5-dicafyloqunic acid ), 4,5-DCQA (4,5-dicafyloqunic acid).

## Supplementary Materials

The Supplementary Materials are available online, Table S1: Desorption stability of protein–phenolic complexes affected by pH and ionic strength.
