# Supplementary material for: Protein–Phenolic Interactions as a Factor Affecting the Physicochemical Properties of White Bean Proteins
Source: Molecules. 2019 Jan 23;24(3):408. doi: 10.3390/molecules24030408 (PMC6384846; doi:10.3390/molecules24030408)
Supplement: Supplementary file 1 [file molecules-24-00408-s001.pdf]

Table S1. Desorption stability of protein–phenolic complexes affected by pH[1] and ionic strength[1].

Sample			Phenolics desorption µmol/g protein ± SD			
			Albumins		Globulins	
			pH 2	1 M NaCl	pH 2	1 M NaCl
Phenolics		GA	$4.8^{Ab}\pm0.49$	$1.8^{Bc}\pm0.44$	$4.5^{Aa}\pm0.34$	$2.9^{Ab}\pm0.64$
		FA	$3.4^{Ac}\pm0.45$	$2.2^{Ac}\pm0.75$	$2.4^{Bb}\pm0.52$	$1.9^{Ab}\pm0.43$
		CGA	$3.6^{Bc}\pm0.25$	$3.2^{Ab}\pm0.53$	$4.7^{Aa}\pm0.80$	$2.2^{Bb}\pm0.26$
		Q	$1.2^{Bd}\pm0.34$	n.d.	$2.5^{Ab}\pm0.24$	$0.8^{Ac}\pm0.23$
		A	n.d.	n.d.	n.d.	n.d.
		CAT	$6.4^{Aa}\pm0.36$	$7.1^{Aa}\pm0.8$	$5.4^{Ba}\pm0.35$	$8.3^{Aa}\pm0.93$
Extracts	GT	GCAT	n.d.	n.d.	n.d.	n.d.
		EGCAT	$3.2^{Ab}\pm0.44$	$0.9^{Bb}\pm0.3$	$2.3^{Bc}\pm0.35$	$1.8^{Ab}\pm0.43$
		CAT	$8.3^{Aa}\pm0.83$	$4.3^{Ba}\pm0.4$	$7.3^{Aa}\pm0.45$	$6.5^{Aa}\pm0.35$
		ECAT	n.d.	n.d.	n.d.	n.d.
		EGCATG	$2.2^{Bc}\pm0.23$	nd	$3.2^{Ab}\pm0.45$	n.d.
		ECATG	n.d.	n.d.	n.d.	n.d.
	GC	NCGA	$0.9^{Ab}\pm0.21$	n.d.	n.d.	n.d.
		CGA	$6.5^{Aa}\pm0.43$	$4.9^{Aa}\pm0.24$	$7.2^{Aa}\pm0.53$	$3.7^{Ba}\pm0.36$
		CCGA	$1.2^{Ab}\pm0.35$	n.d.	n.d.	n.d.
		FQA	n.d.	n.d.	n.d.	n.d.
		3,4-DCQA	n.d.	n.d.	n.d.	n.d.
		3,5-DCQA	n.d.	n.d.	n.d.	n.d.
		4,5-DCQA	n.d.	n.d.	n.d.	n.led.

[1] stability of protein–phenolic complexes was also determined in pH 7 and 10 as well as in the presence of 0M NaCl; however, in these conditions, the analyzed compounds were not detected and the results are not presented in the table. Means (± SD, n = 3) with different lowercase letters superscript within the same column for phenolics, GT extracts, and GC extracts are significantly different (α = 0.05). Means with different bolded uppercase and normal letters superscript within a same row for pH 2 and ionic strength in the presence of 1 M NaCl are significantly different (α = 0.05), respectively. GA, FA, CGA, Q, A, CAT–gallic acid, ferulic acid, chlorogenic acid, quercetin, apigenin, catechin, respectively. Green tea (GT) compounds: GCAT ((−)-gallocatechin), EGCAT ((−)-epigallocatechin), CAT ((+)-catechin), ECAT ((−)epicatechin), EGCATG ((−)-epigallocatechin-3-gallate), and (−)-epicatechin gallate (ECATG); Green coffee (GC) compounds: NCGA (neochlorogenic acid), CGA (chlorogenic acid), CCGA (cryptochlorogenic acid), FQA (3-feruloylquinic acid), 3,4-DCQA (3,4-dicafyloqunic acid), 3,5-DCQA (3,5-dicafyloqunic acid), 4,5-DCQA (4,5-dicafyloqunic acid).
